# Supplementary material for: CD14 Is Involved in the Interferon Response of Human Macrophages to Rubella Virus Infection
Source: Biomedicines. 2022 Jan 26;10(2):266. doi: 10.3390/biomedicines10020266 (PMC8869353; doi:10.3390/biomedicines10020266)
Supplement: Supplementary file 1 [file biomedicines-10-00266-s001.zip › biomedicines-1543175-supplementary.pdf]

Supplementary data

CD14 Is Involved in the Interferon Response of Human Macrophages to Rubella Virus Infection

Erik Schilling[1]; Lukas Pfeiffer[2]; Sunna Hauschildt[3,4]; Ulrike Koehl[1,4,5] and Claudia Claus[6,*]

[1] Institute of Clinical Immunology, Medical Faculty, Leipzig University, Johannisallee 30, 04103 Leipzig, Germany; erik.schilling@medizin.uni-leipzig.de
[2] Institute of Medical Microbiology and Virology, Medical Faculty, Leipzig University, Johannisallee 30, 04103 Leipzig, Germany; lukaspfeiffer05@gmail.com
[3] Institute of Biology, Faculty of Life Sciences, Leipzig University, Talstrasse 33, 04103 Leipzig, Germany; shaus@rz.uni-leipzig.de
[4] Fraunhofer Institute for Cell Therapy and Immunology, Leipzig, Germany;
[5] Institute for Cellular Therapeutics, Hannover Medical School, Hannover, Germany; Ulrike.Koehl@medizin.uni-leipzig.de
[6] Institute of Medical Microbiology and Virology, Medical Faculty, Leipzig University, Johannisallee 30, 04103 Leipzig, Germany; claudia.claus@medizin.uni-leipzig.de

* Correspondence: claudia.claus@medizin.uni-leipzig.de; Tel.: +49-341–9714321

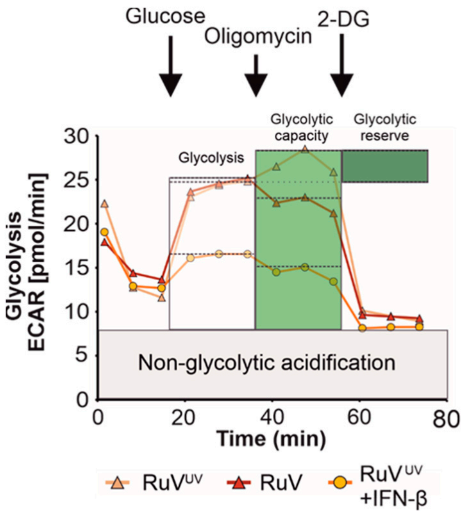

Supplement Figure S1. Representative illustration of the measurement conditions during glycolysis stress test. After glucose starvation for 1 hour, sequential injection of glucose and the inhibitors oligomycin and 2-DG were used to determine glycolytic activity, capacity and reserve as indicated. Shown is a representative measurement graph for indicated samples.

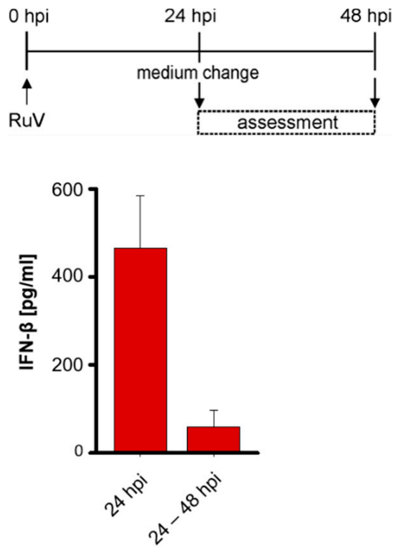

Supplement Figure S2. RuV-induced IFN-β expression in M-MΦ. M-MΦ ($5x10^5$/ml) were infected with RuV. IFN protein levels were determined by LEGENDPLEX human IFN panel kit at 24 hpi and after a medium change at 24 hpi followed by an additional incubation period for 24 hours (n = 3, shown are the means ± SD).
